# Assessment of global reporting of adverse drug reactions for anti-malarials, including artemisinin-based combination therapy, to the WHO Programme for International Drug Monitoring

**DOI:** 10.1186/1475-2875-10-57

**Published:** 2011-03-09

**Authors:** Andrea Kuemmerle, Alex NO Dodoo, Sten Olsson, Jan Van Erps, Christian Burri, Paul S Lalvani

**Affiliations:** 1 Swiss Tropical and Public Health Institute, Department of Medicines Research, Basel, Switzerland; 2 University of Basel, Switzerland; 3 Center for Health Studies, CRP-Santé, Strassen, Luxembourg; 4 Centre for Tropical Clinical Pharmacology & Therapeutics, University of Ghana Medical School, PO Box 4236, Accra, Ghana; 5 WHO Collaborating Centre for International Drug Monitoring (UMC), Box 1051, S-751 40 Uppsala, Sweden; 6 Roll Back Malaria Partnership Secretariat, 20, Avenue Appia, 1211 Geneva 27, Switzerland; 7 RaPID Pharmacovigilance Program, O3i Inc (NGO), PO Box 136, Edgewater, NJ 07020, USA

## Abstract

**Background:**

In spite of enhanced control efforts, malaria remains a major public health problem causing close to a million deaths annually. With support from several donors, large amounts of artemisinin-based combination therapy (ACT) are being deployed in endemic countries raising safety concerns as little is known about the use of ACT in several of the settings where they are deployed. This project was undertaken to profile the provenance of the pharmacovigilance reporting of all anti-malarials, including ACT to the WHO adverse drug reaction (ADR) database (Vigibase™) over the past 40 years.

**Methods:**

The WHO Programme for International Drug Monitoring, the Uppsala Monitoring Centre (UMC) provided anonymized extracts of Vigibase™ covering the period 1968-2008. All countries in the programme were clustered according to their malaria control phase and income status. The number of individual case safety reports (ICSRs) of anti-malarials was analyzed according to those clusters.

**Results:**

From 1968 to 2008, 21,312 ICSRs suspecting anti-malarials were received from 64 countries. Low-income countries, that are also malaria-endemic (categorized as priority 1 countries) submitted only 1.2% of the ICSRs. Only 60 out of 21,312 ICSRs were related to ACT, 51 of which were coming from four sub-Saharan African countries. Although very few ICSRs involved artemisinin-based compounds, many of the adverse events reported were potentially serious.

**Conclusions:**

This paper illustrates the low reporting of ADRs to anti-malarials in general and ACT in particular. Most reports were submitted by non-endemic and/or high-income countries. Given the current mix of large donor funding, the insufficient information on safety of these drugs, increasing availability of ACT and artemisinin-based monotherapies in public and private sector channels, associated potential for inappropriate use and finally a pipeline of more than 10 new novel anti-malarials in various stages of development, the presence of well functioning national pharmacovigilance systems is vital to ensure safe and responsible scale up of ACT deployment. Bringing together the competencies of national pharmacovigilance centres and various types of organizations in the NGO, academic and private sectors with global coordination to create short- and long-term solutions may help address the lag between rapidly growing ACT use and poor ADR reporting.

## Background

In spite of enhanced control efforts, malaria continues to be a major public health problem in 108 countries mostly in Africa and parts of Asia. In 2008, approximately 243 million people fell sick with malaria, with a majority of cases in the African region (85%), causing the death of nearly one million people, 85% of whom were children under five years of age [1]. Malaria and poverty are connected. In the countries where malaria is impacting on public health, it is also severely hampering economic development [2]. As a consequence of increasing resistance of the malaria parasite to previously effective monotherapies including chloroquine (CQ) and sulphadoxine-pyrimethamine (SP), the World Health Organization (WHO) held a technical consultation in 2001 endorsing the potential of artemisinin-based combination therapy (ACT) for drug-resistant malaria [3]. By 2009, most malaria-endemic countries had introduced ACT in their national drug policy, as first-line treatment for uncomplicated *Plasmodium falciparum *malaria [1]. In addition to the ACT, there is a pipeline of more than 10 new anti-malarials in various stages of development which will bring several new therapeutic options to the market in the coming decade [4].

Malaria control programmes are being scaled up with large amounts of international funding commitments that have increased from US$ 0.3 billion in 2003 to US$ 1.7 billion in 2009 thanks to agencies like the Global Fund to fight AIDS, Tuberculosis and Malaria (Global Fund), the US President's Malaria Initiative and the World Bank, as well as with domestic resources. This has allowed increased availability of ACT for public sector use amounting an estimated 240 million ACT distributed from 2004 to 2008 by national programmes [1]. In addition to the public sector channels, ACT and artemisinin-based monotherapies are also available through formal private health channels and also through informal outlets where spurious and substandard drugs are more likely to be found. Due to the high cost of ACT and in a bid to increase access to these medicines, especially for the poor and vulnerable, the Affordable Medicines Facility - malaria (AMFm) of the Global Fund and other partners have mobilized huge financial resources to co-pay the ACT ordered by public and private wholesalers so that these drugs can be made available at prices similar to those of CQ and SP [5,6]. The utilization of anti-malarial treatments in poor and vulnerable populations is common. A large amount of these patients use these drugs in an unsupervised manner which may lead to a significant number of incorrectly treated cases [7-9]. An inappropriate treatment, incorrect dosing, drug-drug interaction, administration in populations suffering from or being treated against concomitant diseases like HIV/AIDS, tuberculosis, malnutrition and anaemia can all impact negatively on drug safety and efficacy [8,10].

At the dawn of global policy change to ACT in early 2000s, safety experience was limited and was mainly derived from clinical research information from South East Asia [11]. In general, safety information can be collected through two main pharmacovigilance channels: (1) spontaneous reporting and (2) systems using pharmacoepidemiological methods through phase IV clinical trials or cohorts [8,12]. While spontaneous reporting is essential for signal detection of rare events, the pharmaco-epidemiological methods provide additional information on both, the utilization and the extent of consumption, that will permit the determination of frequency of adverse drug reaction (ADR) in the studied population or the safety comparison between two or more products [12]. Although ACT are generally considered safe, there is still little structured information about their use in real-life settings and the published data are mainly from clinical trials [13]. Lack of resources, infrastructure and expertise are the main reasons for the slow development of pharmacovigilance systems in developing countries, particularly in sub-Saharan Africa [10,14].

**Figure 1:**
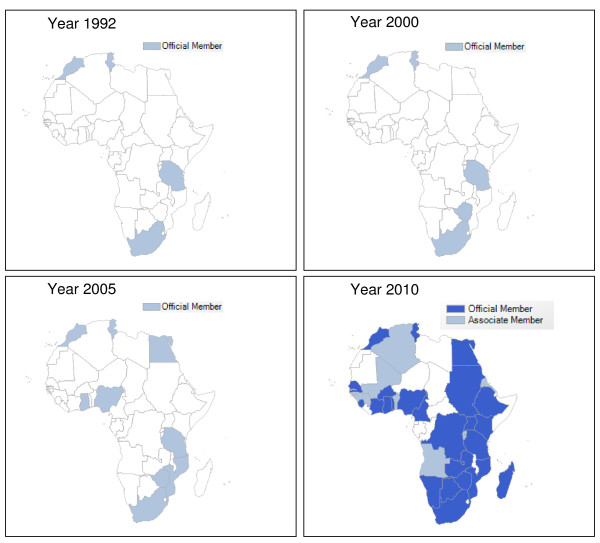
**African WHO members of the programme for international drug monitoring between 1992 and 2010**.

At the global level, the WHO Collaborating Centre for International Drug Monitoring, the Uppsala Monitoring Centre (UMC), collates ADRs via national pharmacovigilance centres of 104 member countries and 32 associate members (December 2010, see Figure 1) [15]. Since the 2000s, WHO and partners have issued a call to establish robust pharmacovigilance systems entailing detection, assessment and prevention of adverse events associated with the use of medicines distributed through the public health system and issued guidelines promoting the development of pharmacovigilance and post-marketing surveillance in 2006 [16]. In that context, the Roll Back Malaria partnership (RBM) suggested that the introduction of ACT could offer an opportunity for African countries to put drug safety monitoring systems in place that could later be extended to a wide range of medicines [17]. Later, RBM issued guidelines to include pharmacovigilance aspects in the Global Fund's Round 8 proposals and any other donors supporting procurement and use of ACT [18,19]. However, despite all those actions implemented since 2000, a recent publication highlighted the lack of consistent inclusion of pharmacovigilance activities in proposals and in country plan components as well as the lack of money requested for such activities in regard to rapid deployment of anti-malarial treatment [20]. It can therefore be expected that spontaneous ADR reporting for anti-malarials is low, especially when focusing to the reports coming from developing and/or endemic countries.

The main objective of the present work was to conduct an analysis of 40 years of ADR reporting of anti-malarials, and to obtain a clear picture on the provenance of the global spontaneous pharmacovigilance data for anti-malarials as well as the identification of the suspected drugs.

## Methods

### Provenance and definition of the dataset

The Uppsala Monitoring Centre (UMC) provided an extract of 40 years Vigibase™ data in the form of a Microsoft^® ^Excel file containing anonymized data on individual case safety reports (ICSR) for all anti-malarial drugs reported to the WHO database since its inception in 1968.

UMC programmed the query that extracted all products which were (1) classified in the ATC system as anti-malarials and (2) considered by the national pharmacovigilance centre submitting the report as suspected or interacting with another drug to cause the ADR. Anti-malarial drugs reported as concomitant medications were not included in the query. The following variables were considered for the present publication: (1) drug name by generic level, (2) report identification number, (3) year of reporting, (4) country issuing the report and (5) system-organ class of reaction reported.

### Data analysis and statistical method

In order to obtain a better understanding of the provenance of the ADR reports, the different countries were clustered according to: (1) their malaria control phase (control, pre-elimination, elimination, prevention of reintroduction or certified malaria free) [1] and (2) their income characteristics as defined by the World Bank (low, middle or high income) [21]. This allowed the classification of the countries into three priority groups according to the matrix presented in Table 1.

**Table 1:** Classification of reporting countries into priority groups

**Malaria control phase **[1]**/Income **[21]	Low income	Middle income	High income
Control	**Priority 1**	Priority 2	*Priority 3*

Pre-elimination/elimination	Priority 2	Priority 2	*Priority 3*

Prevention of reintroduction/Malaria free	*Priority 3*	*Priority 3*	*Priority 3*

The suspected therapies were clustered into three therapeutic groups: (1) Therapy without artemisinin derivatives (WAD), (2) Artemisinin-based monotherapy (AMT) and (3) Artemisinin-based combination therapy (ACT). The data were imported into a Microsoft^® ^Access (2002) database and then extracted with Microsoft^® ^Excel pivot tables (2002). The results are presented as the number of data entries per subgroup (n), the total number of data entries (N) and their frequency expressed as percentage (P % = n/N × 100). The statistical evaluation is descriptive only.

## Results

### General findings

**Figure 2:**
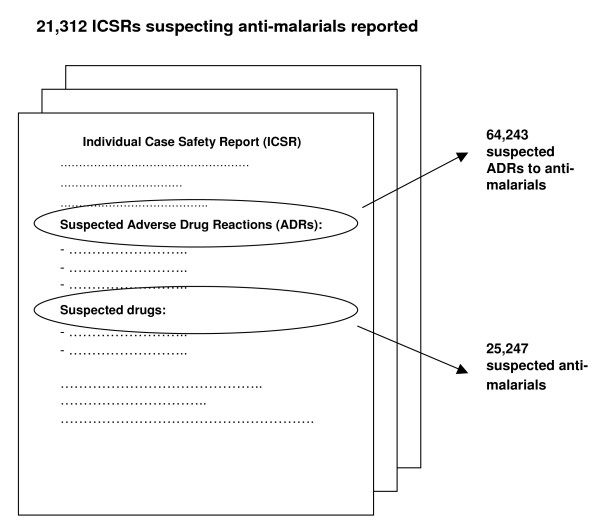
**Individual Case Safety Reports (ICSR) suspecting anti-malarials submitted to UMC from 1968 to 2008**.

64 countries transmitted 21,312 individual case safety reports (ICSR) to UMC from 1968 to 2008, which included a total of 25,247 suspected anti-malarial therapies with a total of 64,243 suspected ADRs to anti-malarials (Figure 2). A mean of three ADRs were described per ICSR. 2,781 ICSRs reported more than one anti-malarial evaluated by the national centre as suspected or interacting in causing the ADR. A mean of 2.4 anti-malarials were incriminated in this subset of ICSRs. 2,027 of those reports contained at least one artemisinin derivative and 1,992 were transmitted by Thailand in the mid-1990s.

### Timeline of reporting

**Figure 3:**
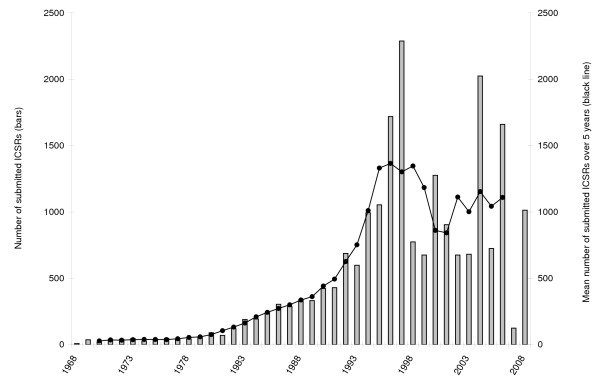
**Number of Individual Case Safety Reports (ICSR) suspecting anti-malarials transmitted to UMC per year (1968 - 2008)**. Note: 21,312 ICSRs have been submitted from 1968 to 2008; in 1997, 1458 of the 2289 reports have been submitted by Thailand; in 2004, 1464 of the 2025 reports have been submitted by the Netherlands

Figure 3 shows the number of ICSRs suspecting anti-malarials transmitted to UMC over time. The first report was received in 1968 and the overall total of ICSRs increased steadily during the 1980s and 1990s and peaked in 1997.

A mean value of 520 ICSRs suspecting anti-malarials were submitted per year to UMC between 1968 and 2008.

### Provenance of the reports

By end 2008, 91 countries were member countries of the UMC and 12 of them were located in sub-Saharan Africa [15]. Approximately 2/3 of the countries worldwide and 1/2 of the countries located in sub-Saharan Africa participating in the UMC programme submitted at least one ICSR suspecting anti-malarials (Table 2).

**Table 2:** List and characteristics of the reporting countries participating to the WHO-UMC programme in 2008 (in brackets: year of entry in the WHO-UMC programme)

**Malaria control phase **[1]**/Income **[21]	Low income	Middle income	High income
Control	**Priority 1**:	**Priority 2**:	
	Ghana (2001)	Brazil (2001)	
	Mozambique (2005)	China (1998)	
	Tanzania (1993)	Colombia (2004)	
	Vietnam (1999)	Costa Rica (1991)	
	Zimbabwe (1998)	India (1998)	
		Indonesia (1990)	
	& 5 countries participating to the UMC programme did not submit any report.	Nigeria (2004)	
		Peru (2002)	
		Philippines (1995)	
		South Africa (1992)	
		Suriname (2007)	
		Thailand (1984)	
		Venezuela (1995)	
		& 3 countries participating to the UMC programme did not submit any report	

Pre-elimination/elimination	2 countries participating to the UMC programme did not submit any report.	**Priority 2**:	1 country participating to the UMC programme did not submit any report.
		Argentina (1994)	
		Iran (1998)	
		Malaysia (1990)	
		Mexico (1999)	
		Turkey (1998)	
		& 1 country participating to the UMC programme did not submit any report.	

Prevention of reintroduction/Malaria free		**Priority 3**:	**Priority 3**:
		Bulgaria (1975)	Australia (1968)
		Chile (1996)	Austria (1991)
		Cuba (1994)	Belgium (1977)
		Lithuania (2005)	Canada (1968)
		Macedonia (2000)	Croatia 1992)
		Morocco (1992)	Cyprus (2001)
		Poland (1972)	Czech Republic (1992)
		Romania (1976)	Denmark (1971)
		Tunisia (1993)	Finland (1974)
		Uruguay (2001)	France (1976)
		Former SFR Yugoslavia	Germany (1968)
			Greece (1990)
		& 11 countries participating to the UMC programme did not submit any report.	Hungary (1990)
			Iceland (1990)
			Ireland (1968)
			Israel (1973)
			Italy (1975)
			Japan (1972)
			Netherlands (1968)
			New Zealand (1968)
			Norway (1971)
			Oman (1995)
			Portugal (1993)
			Singapore (1993)
			Slovakia (1993)
			Spain (1984)
			Sweden (1968)
			Switzerland (1991)
			United Kingdom (1968)
			United States (1968)
			& 5 countries participating to the UMC programme did not submit any report.

Table 3 describes the numbers and percentages of ICSRs suspecting anti-malarials submitted to UMC clustered according to the three country priority groups: high (priority 1), medium (priority 2) and low (priority 3). The reporting rates per million of inhabitants and per thousand of malaria cases are also shown herein.

**Table 3:** Numbers, percentages and reporting rates of Individual Case Safety Reports (ICSR) suspecting anti-malarials submitted to WHO-UMC, clustered according to the country priority groups

	Priority 1 countries	Priority 2 countries	Priority 3 countries	All reporting countries
Number of ICSRs submitted	255	2,983	18,074	21,312

% of total reports	1.2%	14.0%	84.8%	100%

Number of ICSRs (2000-2008)	247	686	8,149	9,082

% of total reports (2000-2008)	2.7%	7.6%	89.7%	100%

Population in thousand (2009) [27]	191,062	3,766,911	1,098,359	5,056,332

Number of ICSRs (2000-2008)/Million population	1.3	0.2	7.4	1.8

Malaria cases in thousand [1]	4,109	3,943	0.2 ^1^	8,052

Number of ICSRs (2000-2008)/thousand malaria case	0.06	0.17	38,439	1.1

^1 ^Malaria cases have been reported by Morocco (118 cases) and Oman (94 cases) [1]

The findings indicate that 89% of reports were submitted by 10, and 97% of reports were submitted by 20 countries, respectively. Only three countries that are in the malaria control phase, Thailand, Ghana and South Africa, rank in the top 20 countries submitting ICSRs involving anti-malarials. Another analysis indicates that sub-Saharan Africa, where 586 million people live in high risk areas and which contributes to 86% of estimated malaria cases and 91% of estimated malaria deaths [22], submitted less than 2% of all ICSRs involving anti-malarials. There were also practically no reports coming from sub-Saharan African countries during 1990s and up to early 2000s until reporting slightly increased. It must be pointed out, however, that during this period, there were just 3 countries from sub-Saharan Africa (South Africa, Tanzania, Zimbabwe) participating in the WHO Programme.

### Suspected anti-malarial therapies reported to UMC

**Figure 4:**
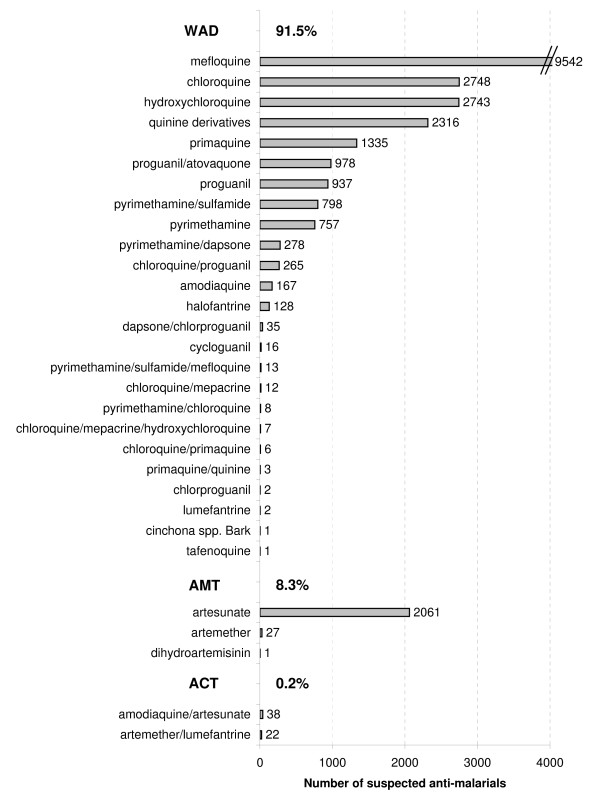
**Number of suspected anti-malarial treatments reported to UMC from 1968 to 2008**. (WAD, Therapy without artemisinin derivatives; AMT, Artemisinin-based monotherapy; ACT, Artemisinin-based combination therapy). Note: 25,247 suspected anti-malarials have been reported from 1968 to 2008

Figure 4 presents the number of suspected (or interacting) anti-malarials, which were reported to WHO-UMC, classified according to their therapeutic group. With a total of 25,247 suspected anti-malarial treatments, the majority of the suspected therapies reported were non-artemisinin compounds (92%) and the minority were ACT (0.2%). Mefloquine was the most reported drug with 79% of mefloquine reports coming from priority 3 countries. Of the AMT reported, Thailand reported 96% of them but, interestingly, did not submit any ICSR suspecting ACT.

**Figure 5:**
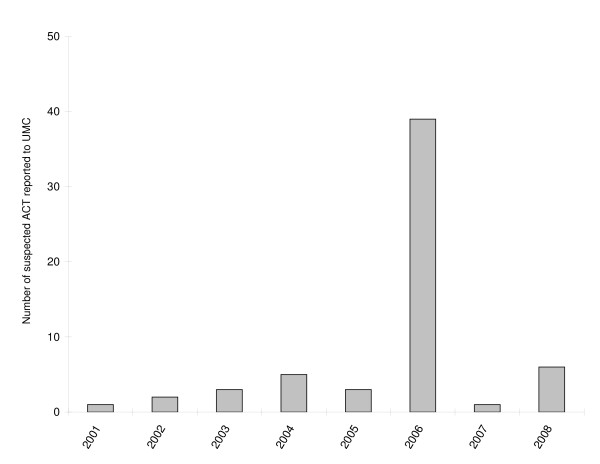
**Number of suspected ACT reported to UMC per year (2001 - 2008)**. (ACT, Artemisinin-based combination therapy). Note: 60 suspected ACT have been reported from 2001 to 2008; in 2006, 37 of the 39 suspected ACT have been reported by Ghana)

The reporting timelines of suspected ACT to UMC are shown in Figure 5. No report suspecting ACT was sent before the WHO recommendation of 2001. The number and percentages of suspected ACT reported to UMC according to the country priority group are listed in Table 4. A total of nine countries transmitted a total of 60 ICSRs suspecting ACT to UMC. From those nine countries, four are still in the malaria control phase and are located in sub-Saharan Africa (Ghana, Nigeria, Tanzania and South Africa) whilst the others are non-endemic (France, Germany, the Netherlands, Switzerland and the United States).

**Table 4:** Number and percentages of suspected ACT reported to UMC clustered according to the country priority groups from 2001 to 2008

	Priority 1 countries	Priority 2 countries	Priority 3 countries	All reporting countries
Number of suspected ACT	40 ^1^	11 ^2^	9 ^3^	60

% of total number of suspected ACT	67%	18%	15%	100%

Note: ACT, Artemisinin-based combination therapy; SSA, sub-Saharan Africa

^1 ^Reports have been submitted by Ghana and Tanzania

^2 ^Reports have been submitted by Nigeria and South Africa

^3 ^Reports have been submitted by France, Germany, the Netherlands, Switzerland and United States

ADRs reported for artemisinin-based compounds, administered either as monotherapy or combination therapy, affected the following 5 most frequently reported MedDRA System Organ Classes: nervous system disorders, gastrointestinal disorders, psychiatric disorders, metabolism and nutrition disorders and cardiac disorders.

## Discussion

This publication explores the scope and scale of spontaneous ADR reporting for anti-malarials and particularly ACT until 2008, with the aim to evaluate and measure the past and current activities in pharmacovigilance for anti-malarials around the world. This work has been accomplished using the largest global database, Vigibase™ of the WHO Programme for International Drug Monitoring maintained by the UMC in Sweden. An extensive description of the nature of the ADRs in relation to the suspected drugs that were reported is planned to be the objective of another publication.

All products that were classified in the ATC system as anti-malarials and considered as suspected or interacting in causing the ADR were extracted from Vigibase™ and included in the present analysis. The assessment of causality between the anti-malarial and the ADR as well as the exact treatment indication, were not included in the query because this information is often incompletely transmitted to UMC. Therefore, drugs that are not classified as anti-malarials in the ATC system but can be used as anti-malarials, like doxycycline, have not been included in the analysis. Conversely, anti-malarials that might have been used for other indications have been included in the present analysis.

Globally, the submission rates of ICSRs suspecting anti-malarials increased until reaching a maximum in 1997 and currently seem to remain stable. The majority of those ICSRs were from priority 3 countries (high income and non-endemic) since the inception of the WHO database in 1968. This trend showing that high income and non-endemic countries are reporting more often has remained stable over the last decade with six to 40 times more ICSRs per inhabitant submitted by high income and non-endemic countries (priority 3) than by endemic and poorer countries (priority 1 and 2). The ICSRs submitted since the year 2000 per malaria case show also that the priority 1 countries, mainly located in sub-Saharan Africa, seem to submit less ICSRs than priority 2 countries. Assuming that patients in high income and/or non-endemic countries are usually taking anti-malarials for travel purposes, the data extracted from the Vigibase™ is probably reflecting the safety of anti-malarials taken as prophylactic rather than as curative treatments. This assumption may be supported by the finding that mefloquine is the most frequently reported anti-malarial and that this compound was extensively used over the years in Northern hemisphere countries for malaria prophylaxis.

Even though ACT is nowadays widely distributed in endemic countries, only 60 ICSRs suspecting ACT have ever been submitted by nine countries (four of them located in sub-Saharan Africa). Those reports were all sent after the WHO recommendation to use ACT for uncomplicated malaria was published in 2001 [3]. Nevertheless, it might actually be that more ICSRs suspecting ACT have been submitted in the mid-1990s. It is unfortunately not possible to know if the 2,027 artemisinin derivatives reported on the same ICSR with other anti-malarials were administered simultaneously as one ACT or separately as one artemisinin-based monotherapy and one non-artemisinin therapy (but within a timeframe allowing the suspicion of both or more drugs). Many of the adverse events reported for ACT or artemisinin-based monotherapy could be potentially serious and involved cardiac, gastro-intestinal, nervous and psychiatric system. At this stage those results should be interpreted cautiously because they may reflect either the ADR profile of artemisinin, the drugs given concomitantly to artemisinin or a combination of both.

The inverse relationship between the huge utilization of anti-malarials and the very low submission rate of ICSRs by priority 1 countries to the WHO drug safety database is coinciding with the fact that sub-Saharan African countries are relatively absent from and/or only recently participating in the WHO Programme for International Drug Monitoring (see Figure 1) [15]. The unacceptably low presence and functioning of national pharmacovigilance systems in sub-Saharan Africa may be due, in addition to lack of human resources, infrastructure and competing priorities, to the absence of earmarked funds and clear guidelines from donor agencies, issues which have been highlighted in a recent publication that examined the status of pharmacovigilance for anti-malarials [20].

The WHO and various donor agencies are currently advocating the development of pharmacovigilance systems in countries where drugs are extensively distributed through public health channels [16,17]. After RBM's call to include pharmacovigilance activities in Global Fund applications followed by further calls from the Global Fund in 2010 for stringent safety monitoring of products, the inclusion of pharmacovigilance as a specific component of the 10^th ^round of the Global Fund, should drive development in a positive direction [18,19,23]. This is important and requires support from all stakeholders to ensure that the safety of ACT as illustrated in the WHO's Vigibase™ is reflective of the countries consuming ACT. Strengthening of spontaneous reporting along with pharmaco-epidemiological methods should provide the needed safety information on ACT, for the benefit of both malaria-endemic countries and the global medical community as a whole. The creation of an office in Africa (Accra, Ghana) by the UMC and the establishment of the University of Ghana Medical School as a WHO Collaborating Centre for Advocacy and Training in Pharmacovigilance [24] are likely to provide rallying points for the mobilization of technical and financial resources for the expansion of pharmacovigilance in Africa.

In order to supplement these long-term capacity building and systems strengthening efforts, the RaPID initiative (Rapid Pharmacovigilance Implementation in Development Countries), is working with public health programs in various countries to provide 'instant' solutions through short workshops and electronic-based support to analyse and submit ICSRs to the WHO database. A WHO-funded study conducted by RaPID highlighted that several countries had collected a large number of ICSR (several hundred), but due to various reasons, were not able to submit these reports to the WHO database [25]. The RaPID initiative includes a consortium of academic, NGO and private sector organizations from developed and developing countries [26].

## Conclusions

This paper shows that there is very low spontaneous reporting of ADRs to anti-malarials in general and ACT in particular, considering the scale of use of anti-malarials globally. Furthermore, the ADR data is being submitted predominantly from non-endemic and/or high-income countries. The reporting figures are even more alarming, when considering that there is practically no reporting of ACT (only 60 reports until end of 2008) while the national programmes reported that an estimated 240 million ACT have been distributed from 2004 to 2008 [1].

It can be inferred that national pharmacovigilance reporting systems are extremely weak or non-existent in a large number of malaria-endemic countries, especially in sub-Saharan Africa. Additional studies will need to be conducted in order to identify the weak links along the pharmacovigilance supply chain, which could be one or more of the following: 1) completion of ICSRs by health workers, 2) analysis and review of the ICSRs by the pharmacovigilance department, 3) submission of ICSRs to UMC, 4) conducting signal detection and strengthening, 5) establishing national policies based on findings and 6) providing feedback to the health workers. In addition, health workers should also be appropriately trained in pharmacovigilance issues and adequate equipment should be provided to them. A large number of patients are treated in rural areas and resource limited settings, such patients will also need to be followed-up in order to detect ADRs in a comprehensive way.

Given the current mix of large funding from donors, the availability of ACT and artemisinin-based monotherapies in public and private sector channels (including informal channels), the significant potential for inappropriate use and overuse, especially in countries where ACT are available over-the-counter, the insufficient information on safety of these drugs in the African context and finally a pipeline of more than 10 novel anti-malarials in various stages of development [4], the presence of active and well functioning national pharmacovigilance systems is vital to ensure safe and responsible scale up of ACT use.

The recent initiative by the Global Fund and WHO to develop a joint pharmacovigilance strategy and a recommendation that Global Fund recipient countries have a minimum capacity of pharmacovigilance systems in place is highly encouraging [16,23]. Bringing together the competencies of national pharmacovigilance centres and organizations in the NGO, academic and private sector with global coordination to create short- and long-term solutions is required to address the lag between rapidly growing ACT use and ensuring public health safety.

## Conflicts of interests

The authors declare no conflicts of interest. ANOD is the Director of the WHO Collaborating Centre for Advocacy and Training in Pharmacovigilance, Accra, Ghana. PSL is the Director of the RaPID Programme. SO is the Chief WHO Programme Officer at the Uppsala Monitoring Centre.

The opinions and conclusions expressed in this article are not necessarily those of the WHO.

## Authors' contributions

PSL conceived the study and organized the data, kindly provided by SO. AK analyzed the data and drafted the manuscript with the help of PSL, ANOD and CHB. All authors reviewed critically the manuscript and approved the final version.
